# Determinants of uptake of intermittent preventive treatment during pregnancy: a review

**DOI:** 10.1186/s12936-019-3004-7

**Published:** 2019-11-21

**Authors:** Elaine Roman, Kristin Andrejko, Katherine Wolf, Marianne Henry, Susan Youll, Lia Florey, Erin Ferenchick, Julie R. Gutman

**Affiliations:** 1 Jhpiego, Baltimore, MD 21231 USA; 2 Division of Epidemiology, School of Public Health, University of California, Berkeley, Berkeley, CA USA; 3 U.S. President’s Malaria Initiative, U.S. Agency for International Development, Washington, DC USA; 4 The Global Fund to Fight AIDS, Tuberculosis and Malaria, Geneva, Switzerland; 5 Malaria Branch, Division of Parasitic Diseases and Malaria, U.S Centers for Disease Control and Prevention, Atlanta, GA USA

**Keywords:** Malaria, Pregnancy, Intermittent preventive treatment, Sulfadoxine–pyrimethamine

## Abstract

Malaria in pregnancy (MiP) contributes to devastating maternal and neonatal outcomes. Coverage of intermittent preventive treatment during pregnancy (IPTp) remains alarmingly low. Data was compiled from MiP programme reviews and performed a literature search on access to and determinants of IPTp. National malaria control and reproductive health (RH) policies may be discordant. Integration may improve coverage. Medication stock-outs are a persistent problem. Quality improvement programmes are often not standardized. Capacity building varies across countries. Community engagement efforts primarily focus on promotion of services. The majority of challenges can be addressed at country level to improve IPTp coverage.

## Background

Annually, approximately 125 million pregnancies occur globally in areas with *Plasmodium falciparum* and/or *Plasmodium vivax* transmission [1].

Malaria in pregnancy (MiP) contributes to devastating maternal and neonatal outcomes, including maternal anaemia, maternal death, stillbirth, spontaneous abortion, and low birth weight, with an estimated 10,000 women and 100,000 infants dying as a result of MiP [2–5]. In sub-Saharan Africa (SSA), MiP contributes to an estimated 20% of all stillbirths and 11% of all newborn deaths [6].

In order to mitigate the consequences of MiP in moderate to high malaria transmission areas, the World Health Organization (WHO) recommends that pregnant women receive intermittent preventive treatment during pregnancy (IPTp) with sulfadoxine–pyrimethamine (SP) [7]. IPTp decreases the incidence of low birth weight by 29%, severe maternal anaemia by 38%, and neonatal mortality by 31% [8, 9]. IPTp is also one of very few health interventions with peer-reviewed evidence demonstrating its impact on reducing neonatal mortality, thereby providing a substantive public health impact in reducing malaria-related mortality [10]. Further, although there is increasing parasite resistance to SP in some areas, IPTp remains a highly cost-effective, lifesaving strategy to prevent the adverse effects of MiP in the vast majority of pregnant women in SSA [11, 12].

In 2012, in response to stagnant coverage rates of IPTp, and in light of new data supporting the use of three or more doses of IPTp, the WHO updated its policy on IPTp [13]. The policy currently promotes the initiation of IPTp-SP in areas of moderate to high malaria transmission, beginning as early as possible in the second trimester, at each scheduled antenatal care (ANC) contact thereafter, at least 1 month apart, until delivery [7]. Use of insecticide-treated bed nets (ITNs) and effective case management continue to be recommended in all areas where pregnant women are at risk for malaria. Achieving high coverage of at least three doses of IPTp (IPTp3) will require delivery of high-quality ANC, as recommended in the updated WHO guidance on ANC, which now promotes a minimum of eight contacts between pregnant women and the health system, as compared to the previously recommended four ANC visits, presenting new opportunities for scaling-up IPTp [14].

**Fig. 1:**
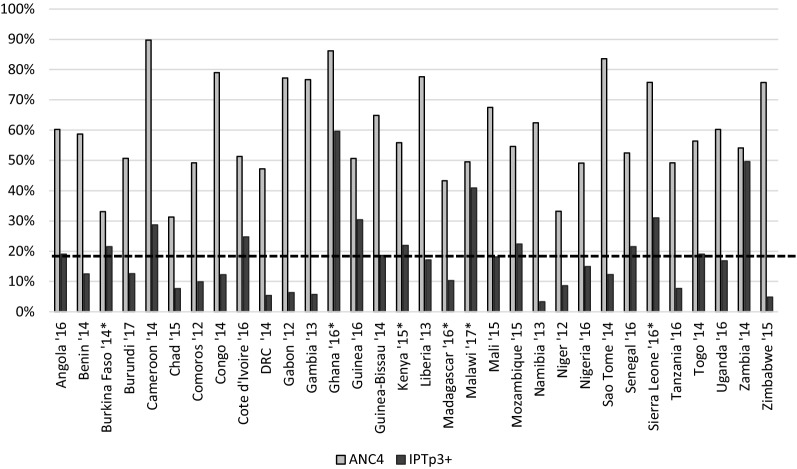
Antenatal Care Visits and IPTp3 Uptake. Data derived from the most recent publicly posted Demographic Health Survey, Malaria Indicator Survey, and Multiple Indicator Cluster Survey (MICS). Unless indicated, ANC and IPTp3+ data were derived from the same survey. For the following, the year indicates the survey where IPTp data were derived, and the ANC data were derived from the survey listed: Burkina Faso 2010 DHS, Ghana 2014 DHS, Kenya 2014 DHS, Madagascar 2012 MICS, and Sierra Leone 2013 DHS. The dotted line represents the overall average of IPTp3+ coverage

As of 2016, 36 African countries had adopted a policy of providing IPTp3 to pregnant women [15]. However, many countries are still far from achieving their targets for IPTp uptake, generally 80% [16]. In 2016, the WHO estimated that coverage of IPTp1, 2, and 3 were 56%, 43%, and 19%, respectively [15]. The gap between high ANC attendance and the low proportion of eligible pregnant women receiving IPTp3 largely reflects a failure of the health system to provide IPTp-SP at ANC facilities (Fig. 1) [17–19]. Drops in coverage between IPTp1 and subsequent doses are particularly concerning [20–30].

With the aim to revitalize a critical evidence-based discussion on strategies to scale-up MiP prevention, published and programmatic literature were searched to assess the continued barriers to IPTp uptake and identify interventions that have been shown to improve IPTp coverage.

## Methods

### Search strategy

Data from country MiP programme reviews, including programme reports, case studies, planning documents, conference presentations, were compiled to better understand what makes MiP programming successful, including what has increased coverage of IPTp, remaining bottlenecks, and opportunities to improve existing efforts. These reviews, examining IPTp uptake in 18 countries from 2009 to 2017, were identified in consultation with key stakeholders supporting MiP programme implementation, including the President’s Malaria Initiative and the Global Fund to Fight AIDS, Tuberculosis and Malaria.

A systematic literature search was performed according to PRISMA guidelines on May 4, 2018 for studies on access and determinants of IPTp use published since May 31, 2016, to provide an update from the most recently published systematic review on this topic [31, 32]. Pubmed and the malaria in pregnancy library (http://library.mip-consortium.org/), a comprehensive database of published and unpublished literature on MiP, were searched for all English language studies looking at access, coverage, feasibility, or acceptability of IPTp-SP among pregnant women in SSA. In addition, the database of the Health Care Provider Performance Review was searched for studies among pregnant women in SSA assessing methods of improving health worker performance and care seeking [33].

### Data analysis

The programme reviews and existing literature were summarized, and key findings synthesized by focusing on seven of the eight key areas necessary for effective MiP programming: (1) policy; (2) integration; (3) commodities; (4) quality improvement; (5) capacity-building; (6) community engagement; and (7) monitoring and evaluation (M&E) [34]. Further, while each core component contributes individually to strengthening MiP programming, there is an integral link between all core components. When one area is weak, it can negatively affect another area, and when an area is strong it can bolster other components [34]. For this review, policy and integration were combined for analysis. The eighth key area, financing, was not addressed.

## Results

**Fig. 2:**
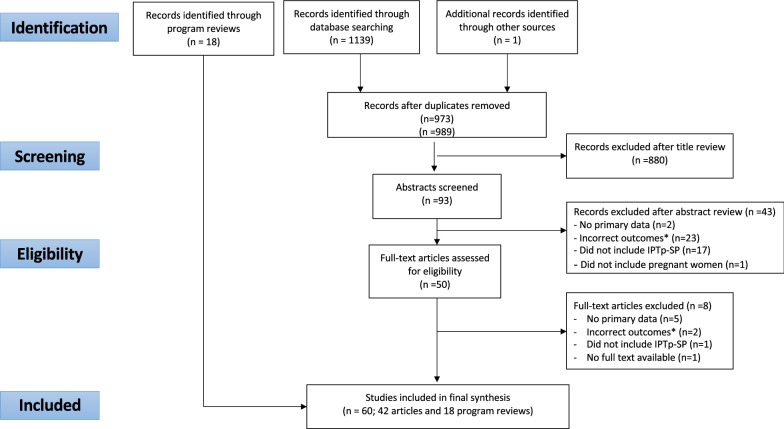
PRISMA flow diagram of studies included in this review. Studies were assessed for eligibility using the PICOTs framework, with Population: Pregnant women, Intervention: IPTp-SP, Outcomes: Access, coverage, feasibility, acceptability, Timing: for peer reviewed literature: May 31, 2016 through May 4, 2018 and for program reviews: no restrictions; Setting: sub-Saharan Africa, Language: English

Following de-duplication, a total of 973 records were identified; after title screening, 93 abstracts and 50 full text articles were reviewed (Fig. 2). Ultimately, 42 unique studies were eligible. Twenty programme reviews were also included.

### Policy and integration

Discordant guidance and poor implementation of national MiP policies between national reproductive health programmes (RHP) and national malaria control programmes (NMCPs) have been observed. A review of policy documents, national guidelines, and training and supervision materials from NMCPs and RHPs in 17 SSA countries in 2014 found that multiple countries were promoting out-of-date guidance with respect to the WHO recommendations [35]. A more recent review in 2018 confirmed that there continue to be significant discrepancies between NMCP and RHP documents [35]. These discrepancies contribute to provider confusion. In Uganda, providers did not feel that national-level documents clearly outlined the dosing schedule for IPTp, and many workers were confused about the timing and dosing of IPTp [36]. Low IPTp coverage in Mali and Tanzania has also been attributed, in part, to confusing policy guidance [37, 38]. Also since, co-administration of co-trimoxazole and SP is contraindicated, there must be strong coordination between NMCP, RHP, and national HIV programmes to ensure pregnant women living with HIV receive optimal care [14]. Beyond harmonization of policies for MiP, national policies that reinforce and support more education for girls may also have a positive impact on IPTp uptake, as it is well documented that girls with a higher education have increased IPTp uptake [28, 30, 39–45]. Moreover, the 2017 WHO ANC recommendations do not explicitly promote the importance of partnership between national RH and national malaria control programmes.

In addition to harmonized policies, multiple countries have also documented how they have created effective integration between programmes to improve MiP outcomes. In Ghana, the NMCP and Reproductive and Child Health Department (RCHD) worked closely together to develop national policy documents, including guidelines and training materials [46]. Between 2006 and 2016, Ghana made impressive gains in increasing uptake of IPTp2, with coverage increasing from 28 to 78%. Between 2014 and 2016, IPTp3 increased from 38 to 60% [47]. In Uganda, the NMCP worked with the RH programme to create a MiP Technical Working Group, which reviewed and updated the necessary policy documents to adopt the 2012 WHO recommendations. From 2011 to 2015, Uganda demonstrated improvements in coverage, with IPTp2 increasing from 25 to 45% and IPTp3 increasing from 9 to 25% [48].

### Commodities

Stock-outs of SP—primarily at ANC facilities and sometimes at the national level—continue to occur across countries, directly affecting IPTp coverage [22, 23, 26, 27, 29, 36, 49, 50]. When stocks of SP are available, however, user fees are cited as an inhibiting factor in many countries [22, 23, 49–51]. Further, when stocks of SP are low at facility level, prescribing practices may deviate from policy and healthcare providers may prioritize giving IPTp1 over IPTp2 [36]. In some cases, women will go to a drug vendor and pay for SP, which is rarely documented in the national health system [20]. Many health care providers and national policymakers have the perception that SP is not an effective drug because of documented resistance to SP in the setting of case management, contributing to the failure of health care providers to administer the drug to pregnant women for IPTp [52]. Lastly, the lack of availability of clean water and cups has been shown to have a negative impact on IPTp uptake [21, 23].

### Quality improvement

Quality of care is defined as the extent to which health care services provided to individuals improve desired health outcomes [53]. In this review, quality includes performance standards to measure and supervision support to improve quality of care and self-assessment by health care providers to self-monitor service delivery. Based on data presented in the case studies, quality improvement to reinforce supervision and mentoring of health care providers varied [54–57]. In most countries, quality improvement systems for service delivery were considered weak, and lack the resources, including trained supervisors, to provide consistent and effective supportive supervision from the district to facility level [54–56]. Case studies from Zambia, Senegal, and Mozambique highlight how all are using standards to help improve the quality of care, which is contributing to improved IPTp uptake [55, 56, 58, 59].

### Capacity-building

While countries generally include capacity-building of the health workforce in their national health sector plans, the nature and frequency of training varies across countries depending on national and external resources. It is not surprising that gaps in provider knowledge and skills to effectively deliver IPTp-SP also vary by country, with notable variation between providers’ knowledge on timing and number of doses of IPTp, timing of first dose of IPTp, and management of stock-outs [22, 24, 36, 49, 60, 61]. Many studies cited a lack of provider training on policies and procedures directly related to current IPTp policy and administration procedures [36, 44, 49, 61]. Focused efforts to improve training in Madagascar, Benin, Tanzania, Kenya, and Nigeria were reported to contribute to increased uptake of IPTp in target areas [58, 59, 62]. A three-day training for healthcare workers on MiP clinical practice coupled with supportive supervision, updated job aids, and community brochures contributed to higher IPTp use compared to study sites without this package in western Kenya [45]. Training to ensure that women are treated respectfully may help improve coverage, as women have reported that disrespectful care prevents them from attending ANC [36]. Women have also reported being turned away by providers if they presented without their husbands, causing them in some cases not to return [36].

### Community engagement

Many countries (e.g., Guinea, Ghana, Zambia, Senegal, and Malawi) have introduced some level of community involvement to improve community education and mobilization for MiP [48, 58, 59]. Promotion of early ANC attendance was documented across several studies to improve IPTp coverage [22, 24, 28, 30, 43, 44, 63–66]. Further, health education mitigates common misconceptions which prevent women from seeking treatment in pregnancy: concerns about using medication in pregnancy, taking drugs on an empty stomach, and fear of adverse effects [49, 51, 67, 68]. Additionally, education of husbands could be an important factor, as one study in Nigeria found IPTp uptake is higher in households where both partners understand benefits of ANC and IPTp [67]. Finally, several countries are currently testing a community delivery approach of IPTp to augment ANC delivery and improve access [69–71].

### M&E

MiP indicators are generally tracked through a variety of sources in malaria-endemic countries, including: periodic household surveys, such as Demographic and Health Surveys (DHS), Multiple Indicator Cluster Surveys (MICS), and Malaria Indicator Surveys (MIS); the routine national Health Management Information System (HMIS); and less commonly, sentinel site surveillance systems [72]. No studies were identified that explicitly addressed M&E for MiP. Denominators used for routine data collection and household surveys are not the same, creating discrepancies in the data, and making it difficult to directly compare reported coverage rates. At the facility level, the denominator used for IPTp uptake in most countries is total number of 1st visit ANC clients, whereas the denominator used for household surveys is total number of surveyed women aged 15–49 with a live birth in the specified time period. The programme reviews cited weak recording and reporting practices, and that programme managers and health care providers rarely use data for decision-making [54–56, 73]. The reviews also suggested that updates to ANC registers and to HMIS data elements and indicators were slow and inconsistently implemented.

## Discussion

Despite lower than desired coverage of MiP interventions overall, countries are taking steps to improve coverage and address bottlenecks.

### Policy

Consistency between national RH and NMCP documents, including guidelines and training materials, sets the stage for effective planning, coordination, and implementation and minimizes confusion among supervisors and health care providers [74]. Consistency in messaging should also be ensured with HIV programmes, as ANC providers deliver all of these services together.

The release of the 2017 WHO ANC recommendations provides a good opportunity to harmonize RH and NMCP policies and guidelines for IPTp, as countries update their national ANC guidelines. However, and additionally, direct guidance from WHO to foster partnership between national RH and national malaria control programs could engender the harmonization process. Interestingly, Zambia and Ghana, whose initial policies recommended at least 3 doses of IPTp, have achieved some of the highest IPTp coverage rates in SSA, which may indicate that a policy promoting frequent dosing creates an enabling environment for better coverage. The 2012 WHO IPTp policy recommendation promotes dosing at each scheduled ANC visit, giving countries a chance to achieve higher coverage of at least three doses. However, effectively integrating this recommendation into the new ANC guidance will require collaboration between the RHP and NMCP to ensure that recommendations are clear and provide for optimal dosing. This includes determining how best to initiate IPTp-SP early in the 2nd trimester, ideally by recommending an ANC contact before 13 weeks, with the first dose of SP given before 13–16 weeks [87].

### Integration

Countries with strong partnerships between and leadership among national RHPs and NMCPs, as well as HIV, generally report better coverage of IPTp. There is a particular need for RHPs and NMCPs to work together to review and address the barriers to achieving IPTp uptake (e.g., inconsistency in guidelines, misunderstanding of guidelines among health workers, lack of supplies, SP stock-outs), set targets, and work together to harness funding for MiP programming through the delivery of RH programmes. Without these strong partnerships in place, MiP may become marginalized within the health system where it is not owned by the RH programme or the NMCP. This can contribute to disjointed policies, duplicative and/or discordant training, and lack of accountability, particularly when RH programmes are continually being asked to do more with little to no additional funding. The reestablishment or strengthening of MiP working groups, bringing together representatives from RHP, NMCP, and other key partners supporting MiP programming, has been previously documented as a best practice to foster integration and harmonization, including prioritization of MiP programming [75, 76].

### Commodities

SP for IPTp continues to be effective [31]. This key message needs to be targeted to policymakers, managers, and health care providers, especially in countries where parasite resistance to SP is high. MiP national technical working groups can play a role in advocating for the correct quantity and quality of SP, as well as reviewing and addressing country procurement and supply chain bottlenecks that affect delivery of SP from the national level to ANC. Further, advocacy around the use of SP only for prevention, not for treatment, may improve coverage of IPTp.

Stock-outs of SP across all levels of the health system, as well as irrational use of SP, contribute to low coverage of IPTp. If SP procurement is not prioritized, emergency procurement may be required, and stock-outs may occur, particularly in light of the long lead time required to procure SP. Even when central stocks are available, many countries report facility level stock-outs, as a result of poor stock management practices. Health system weaknesses such as the capacity/ability of ANC facilities to obtain SP from the district level are persistent in many countries. Consistent availability of SP in countries, as well as at point of care, is paramount to increase coverage of IPTp.

### Quality improvement

There was a paucity of data in the available peer reviewed literature that related quality improvement systems to IPTp outcomes. Rowe et al. reported that the development of management tools for effective resource allocation will increase health workers performance and improve general health outcomes [77]. Efforts to strengthen health worker management and performance will likely positively influence IPTp uptake. Giving providers and onsite managers the tools to self-assess, identify bottlenecks (e.g. care, facility readiness), and address performance gaps immediately could help to improve provider practices and ultimately coverage [78]. Further, harmonizing health facility performance standards with national guidelines and training materials could help to reinforce providers’ knowledge and skills. Empowering health care providers and onsite managers to self-assess and address gaps in care leads to self-motivation and improved quality of care [79].

### Capacity-building

Effectiveness of training depends on many variables, including the capacity of trainers, the content of the material, and the prioritization to continually build providers’ knowledge and skills through supportive supervision. If trainers do not have the skills to transfer knowledge and skills adequately, the trainees’ ability to absorb what they are learning is minimized. Further, if training materials are not aligned with national policies, and harmonized between the RHP and NMCP, providers can be confused. Finally, one-time trainings alone may be insufficient [77]. To maximize learning content, longitudinal, integrated training approaches should be coupled with quality improvement mechanisms, as described earlier. Further, trainees should be provided with written materials and job aids to help reinforce materials covered in training, although even the combination of training and job aids may only have a modest effect in absence of quality supervision [45, 74]. Prioritization of pre-service education is key to ensuring that graduates entering the workforce have the capacity and confidence to support the comprehensive needs of women attending ANC [80]. Capacity building for improving IPTp uptake should include training in early gestational age assessment, as there are reports that women who present very early are turned away and told to come back when they are “showing” [81]. However, gestational age can be determined by symphysis-fundal height (SFH) starting at approximately 12 weeks of gestational age, when the fundal reaches the level of the symphysis pubis. Symphysis-fundal height measurements have been demonstrated to increase linearly with gestational age [82]. Thus, palpation of the uterine fundus can be used to assist in assessing when it is safe to initiate IPTp.

### Community engagement

While community interventions can successfully address both supply and demand-side issues, countries have slowly begun to incorporate and prioritize community engagement that promotes, and in some cases, delivers IPTp. Frequently, women do not understand when, or even that, they should be taking IPTp-SP [20, 23–27, 30, 50, 64, 83–87]. Community engagement is important to ensure that both pregnant women and their partners understand the importance of taking IPTp and starting it early in pregnancy, and are able to demand IPTp to ensure that optimal coverage is achieved. If they are unaware of the benefits of IPTp, they will not seek it out nor ask for it when they attend ANC [26, 51]. The new WHO ANC recommendations underscore the importance of care at the community level, including early promotion of ANC contact at either the facility or community level. Community care may offer a critical opportunity for countries to maximize coverage for pregnant women [88].

### Monitoring and evaluation

Countries routinely collect data on IPTp uptake, and IPTp coverage is regularly measured in national-level health surveys. However, given that routine data collection and national level surveys are using different denominators, this can lead to confusion and an inability to prioritize interventions correctly to address gaps in care or optimize better practices. In addition, the rapid development of routine health information systems means that the completeness and quality of the data being reported has changed greatly over time and is therefore difficult to interpret [15].

Standardization of data elements and of indicators for routine data would help interpretation across settings and over time. Using the revised surveillance, monitoring and evaluation (SM&E) guidance from WHO will likely help to ensure that data are collected in a uniform fashion across countries and facilitate comparisons [89]. A greater investment in M&E and prioritizing a data for decision making are needed to make the best use of available data. Finally, it is important to note that to ascertain the correct denominator for IPTp coverage indicators, HIV-positive women currently on co-trimoxazole prophylaxis should be routinely excluded from the total number of IPT-eligible women [19].

## Limitations

This review has several limitations to note. First, there is paucity of data on the impact of MiP integration, quality improvement, and M&E limiting the ability to draw robust conclusions for these areas. Second, the most detailed available programme reviews were conducted over 5 years ago and perhaps some of the details of programmatic evidence is now outdated. Furthermore, programme reviews, in general, are somewhat subjective and only reveal findings based on country reports and/or the persons interviewed. Finally, this review focused on the public sector, given that so little has been documented about MiP programming in the private sector. Still, this review identified barriers to IPTp coverage and identified some interventions that may help to improve IPTp coverage in SSA.

## Conclusions

Although there is a need for continued progress with prevention efforts, the successes of MiP programming across certain countries present an opportunity to identify intervention that can improve IPTp coverage. Strengthening existing RH platforms to deliver services, including IPTp, to eligible pregnant women is essential to maximize opportunities for these women to receive at least three doses of IPTp.

As a maternal and newborn health issue, MiP programming will not succeed without RH leadership. All countries have health system challenges to varying degrees. Addressing these, and tackling IPTp uptake through a health system lens, will lead to better outcomes across countries. This requires tackling all key areas for MiP (policy, integration, commodities, quality improvement, capacity-building, community engagement, and M&E)—these are interdependent, thus, when one is not functioning or is weak, it will negatively affect the entire system and the ability for countries to achieve their results.

## Data Availability

All of the data presented here are published.

## Abbreviations

ANC: antenatal care
DHS: Demographic in Health Survey
HMIS: Health Management Information System
IPTp: intermittent preventive treatment in pregnancy
ITN: insecticide-treated net
M & E: monitoring and evaluation
MICS: Multiple Indicator Cluster Survey
MiP: malaria in pregnancy
MIS: Malaria Indicator Survey
NMCP: National Malaria Control Programme
RCHD: Reproductive Child Health Department
RH: reproductive health
SP: sulfadoxine–pyrimethamine
SSA: sub Saharan Africa
WHO: World Health Organization

## References

[1] Dellicour S, Tatem AJ, Guerra CA, Snow RW, ter Kuile FO (2010). Quantifying the number of pregnancies at risk of malaria in 2007: a demographic study. PLoS Med..

[2] Fleming AF (1989). Tropical obstetrics and gynaecology. 1. Anaemia in pregnancy in tropical Africa. Trans R Soc Trop Med Hyg..

[3] Steketee RW, Wirima JJ, Slutsker L, Heymann DL, Breman JG (1996). The problem of malaria and malaria control in pregnancy in sub-Saharan Africa. Am J Trop Med Hyg.

[4] WHO (2004). Strategic framework for malaria prevention and control during pregnancy in African Region, AFR/MAL/04/01.

[5] Guyatt H, Snow R (2004). Impact of malaria during pregnancy on low birth weight in sub-Saharan Africa. Clin Microbiol Rev.

[6] Eisele TP, Larsen DA, Anglewicz PA, Keating J, Yukich J, Bennett A (2012). Malaria prevention in pregnancy, birthweight, and neonatal mortality: a meta-analysis of 32 national cross-sectional datasets in Africa. Lancet Infect Dis..

[7] WHO (2014). Policy brief for the implementation of intermittent preventive treatment of malaria in pregnancy using sulfadoxine–pyrimethamine (IPTp-SP).

[8] Garner P, Gulmezoglu A (2006). Drugs for preventing malaria in pregnant women. Cochrane Database Syst Rev..

[9] Bhutta ZA, Das JK, Bahl R, Lawn JE, Salam RA, Paul VK (2014). Can available interventions end preventable deaths in mothers, newborn babies, and stillbirths, and at what cost?. Lancet.

[10] Menendez C, Bardaji A, Sigauque B, Sanz S, Aponte JJ, Mabunda S (2010). Malaria prevention with IPTp during pregnancy reduces neonatal mortality. PLoS ONE.

[11] Sicuri E, Bardaji A, Nhampossa T, Maixenchs M, Nhacolo A, Nhalungo D (2010). Cost-effectiveness of intermittent preventive treatment of malaria in pregnancy in southern Mozambique. PLoS ONE.

[12] Flegg JA, Patil AP, Venkatesan M, Roper C, Naidoo I, Hay S (2013). Spatio-temporal mathematical modelling of mutations of the *dhps* gene in African *Plasmodium falciparum*. Malar J..

[13] Kayentao K, Garner P, van Eijk AM, Naidoo I, Roper C, Mulokozi A (2013). Intermittent preventive therapy for malaria during pregnancy using 2 vs 3 or more doses of sulfadoxine–pyrimethamine and risk of low birth weight in Africa: systematic review and meta-analysis. JAMA.

[14] WHO (2016). Recommendations on antenatal care for a positive pregnancy experience.

[15] WHO (2017). World malaria report.

[16] PMI. Angola: malaria operational plan FY. 2018.

[17] Pell C, Menaca A, Afrah NA, Manda-Taylor L, Chatio S, Were F (2013). Prevention and management of malaria during pregnancy: findings from a comparative qualitative study in Ghana, Kenya and Malawi. Malar J..

[18] WHO (2015). World malaria report 2015.

[19] Andrews KG, Lynch M, Eckert E, Gutman J (2015). Missed opportunities to deliver intermittent preventive treatment for malaria to pregnant women 2003–2013: a systematic analysis of 58 household surveys in sub-Saharan Africa. Malar J..

[20] Ameh S, Owoaje E, Oyo-Ita A, Kabiru CW, Akpet OEO, Etokidem A (2016). Barriers to and determinants of the use of intermittent preventive treatment of malaria in pregnancy in Cross River State, Nigeria: a cross-sectional study. BMC Pregnancy Childbirth..

[21] Doku DT, Zankawah MM, Adu-Gyamfi AB (2016). Factors influencing dropout rate of intermittent preventive treatment of malaria during pregnancy. BMC Res Notes..

[22] Hurley EA, Harvey SA, Rao N, Diarra NH, Klein MC, Diop SI (2016). Underreporting and missed opportunities for uptake of intermittent preventative treatment of malaria in pregnancy (IPTp) in Mali. PLoS ONE.

[23] Muhumuza E, Namuhani N, Balugaba BE, Namata J, Ekirapa Kiracho E (2016). Factors associated with use of malaria control interventions by pregnant women in Buwunga subcounty, Bugiri District. Malar J..

[24] USAID, PMI. Facility level factors influencing the uptake of intermittent preventative therapy for malaria in pregnant women. Report on a formative assessment conducted in Uganda. African Strategies for Health. 2016.

[25] Nsibu C, Manianga C, Kapanga S, Mona E, Pululu P, Aloni M (2016). Determinants of antenatal care attendance among pregnant women living in endemic malaria settings: experience from the Democratic Republic of Congo. Obstet Gynecol Int..

[26] Protas J, Tarimo D, Moshiro C (2016). Determinants of timely uptake of ITN and SP (IPT) and pregnancy time protected against malaria in Bukoba, Tanzania. BMC Res Notes..

[27] Ayubu MB, Kidima WB (2017). Monitoring compliance and acceptability of intermittent preventive treatment of malaria using sulfadoxine pyrimethamine after ten years of implementation in Tanzania. Malar Res Treat..

[28] Olukoya O, Adebiyi O (2017). Missed opportunities for intermittent preventive treatment for malaria in pregnancy in Nigeria: evidence from demographic and health survey in Nigeria 2013. Ann Ib Postgrad Med..

[29] Salomão C, Sacarlal J, Gudo ES (2017). Assessment of coverage of preventive treatment and insecticide-treated mosquito nets in pregnant women attending antenatal care services in 11 districts in Mozambique in 2011: the critical role of supply chain. Malar J..

[30] Arnaldo P, Rovira-Vallbona E, Langa JS, Salvador C, Guetens P, Chiheb D (2018). Uptake of intermittent preventive treatment and pregnancy outcomes: health facilities and community surveys in Chókwè district, southern Mozambique. Malar J..

[31] Desai M, Hill J, Fernandes S, Walker P, Pell C, Gutman J (2018). Prevention of malaria in pregnancy. Lancet Infect Dis..

[32] Liberati A, Altman DG, Tetzlaff J (2009). The PRISMA statement for reporting systematic reviews and meta-analyses of studies that evaluate healthcare interventions: explanation and elaboration. BMJ.

[33] Rowe A, Rowe SY, Peters DH, Holloway KA, Ross-Degnan D. Update of a systematic review of the effectiveness of strategies to improve health care provider practices in low- and middle-income countries. Under Review. 10.1016/S2214-109X(18)30398-X PMC6185992 30309799

[34] Roman E, Wallon M, Brieger W, Dickerson A, Rawlins B, Agarwal K (2014). Moving malaria in pregnancy programs from neglect to priority: experience from Malawi, Senegal, and Zambia. Glob Health Sci Pract..

[35] USAID. PMI. Review of national-level malaria in pregnancy documents in 19 PMI focus countries. Maternal and Child Health Integrated Program, Jhpiego Publ., Baltimore. 2014. https://www.mchip.net/sites/default/files/mchipfiles/19%20Country%20Review%20of%20MIP.pdf. Accessed 15 July 2018.

[36] Rassi C, Graham K, Mufubenga P, King R, Meier J, Gudoi SS (2016). Assessing supply-side barriers to uptake of intermittent preventive treatment for malaria in pregnancy: a qualitative study and document and record review in two regions of Uganda. Malar J..

[37] Mubyazi GM, Bloch P (2014). Psychosocial, behavioural and health system barriers to delivery and uptake of intermittent preventive treatment of malaria in pregnancy in Tanzania—viewpoints of service providers in Mkuranga and Mufindi districts. BMC Health Serv Res..

[38] Webster J, Kayentao K, Diarra S, Diawara SI, Haiballa AA, Doumbo OK (2013). A Qualitative health systems effectiveness analysis of the prevention of malaria in pregnancy with intermittent preventive treatment and insecticide treated nets in Mali. PLoS ONE.

[39] Ghansah G (2016). Factors promoting and preventing the utilization and uptake of ipt among pregnant women in the mampong municipality, Ghana.

[40] Mallick L, Winter R, Wang W, Yourtkavitch J. Integration of infectious disease services with antenatal care services at health facilities in Kenya, Malawi, and Tanzania. DHS Analytical Studies, 62; 2016.

[41] Yusuf O, Akinyemi J, Fagbamigbe A, Ajayi I, Bamgboye E, Ngige E (2016). Controlling malaria in pregnancy: how far from the Abuja targets?. MalariaWorld J..

[42] Tobin-West C, Kanu E (2016). Factors influencing the use of malaria prevention methods among women of reproductive age in peri-urban communities of Port Harcourt City, Nigeria. Niger Postgrad Med J..

[43] Dionne-Odom J, Westfall AO, Apinjoh TO, Anchang-Kimbi J, Achidi EA, Tita ATN (2017). Predictors of the use of interventions to prevent malaria in pregnancy in Cameroon. Malar J..

[44] Awantang GN, Babalola SO, Koenker H, Fox KA, Toso M, Lewicky N (2018). Malaria-related ideational factors and other correlates associated with intermittent preventive treatment among pregnant women in Madagascar. Malar J..

[45] Ouma PO, Van Eijk AM, Hamel MJ, Sikuku E, Odhiambo F, Munguti K (2007). The effect of health care worker training on the use of intermittent preventive treatment for malaria in pregnancy in rural western Kenya. Trop Med Int Health..

[46] USAID, PMI. Successful practices to increase intermittent preventive treatment in Ghana: Program brief. Maternal and child survival program, Jhpiego Publ., Baltimore, 2015.

[47] PMI. Ghana: Malaria Operational Plan FY2018. https://www.pmi.gov/docs/default-source/default-document-library/malaria-operational-plans/fy-2018/fy-2018-ghana-malaria-operational-plan.pdf?sfvrsn=5.

[48] PMI. 10th Annual Report, 2016. https://www.pmi.gov/docs/default-source/default-document-library/pmi-reports/pmi-tenth-annual-report-congress.pdf.

[49] Rassi C, Siduda SG, Graham K, Meier J, Ssekitoleeko J, Drile LV, et al. Assessing and addressing barriers to IPT2 uptake in Uganda. Malaria Consortium. 2016. https://assets.publishing.service.gov.uk/media/5ae3309740f0b631578aef07/Assessing-and-addressing-barriers-to-IPT2-uptake-in-Uganda-policy-brief.pdf.

[50] Okoronkwo I, Okoye H (2016). Factors influencing utilization of intermittent preventive treatment and long lasting insecticide treated bed nets by pregnant women in Anambra State, Nigeria. Int J Adv Sci Res Manag..

[51] Rassi C, Graham K, King R, Ssekitooleko J, Mufubenga P, Gudoi SS (2016). Assessing demand-side barriers to uptake of intermittent preventive treatment for malaria in pregnancy: a qualitative study in two regions of Uganda. Malar J..

[52] ACTwatch. SP availability and (mis) use in sub-Saharan Africa: Antimalarial market data from 8 countries. (Poster). 64th Annual Society of Tropical Medicine and Hygiene Conference, 2015.

[53] WHO. Maternal, newborn, child and adolescent health. Geneva: World Health Organization; 2018. http://www.who.int/maternal_child_adolescent/topics/quality-of-care/en/. Accessed 15 Aug 2018.

[54] Sethi R, Seck A, Dickerson A. A malaria in pregnancy country case study: Senegal’s successes and remaining challenges for malaria in pregnancy programming. USAID: PMI, 2011.

[55] Wallon M, Agarwal S, Roman E, Dickerson A. A malaria in pregnancy country case study: Malawi’s successes and remaining challenges for malaria in pregnancy programming. USAID: PMI, 2011.

[56] Wallon M, Roman E, Brieger W, Rawlins B. A malaria in pregnancy case study: Zambia’s successes and remaining challenges for malaria in pregnancy programming. USAID: PMI, 2010.

[57] Udu R, Ochieng D, Apat D (2017). The level of adoption of malaria prevention strategies on pregnant women in Sauri, Siaya sub county, Kenya. Int J Adv Sci Res Manag..

[58] PMI. 8th Annual Report, 2014. https://www.pmi.gov/docs/default-source/default-document-library/pmi-reports/pmireport_final.pdf?sfvrsn=18.

[59] PMI. 9th Annual Report, 2015. https://www.pmi.gov/docs/default-source/default-document-library/pmi-reports/pmi-ninth-annual-report-congress.pdf?sfvrsn=11.

[60] Oranu EO, Ojule JD, Ordu JS (2016). Malaria chemoprophylaxis during pregnancy: a survey of current practice amongst general practitioners in Port Harcourt, Nigeria. Port Harcourt Med J..

[61] Stephen A, Wurapa F, Afari EA, Sackey SO, Malm KL, Nyarko KM (2016). Factors influencing utilization of intermittent preventive treatment for pregnancy in the Gushegu district, Ghana, 2013. Pan Afr Med J..

[62] PMI. 11th Annual Report, 2017. https://www.pmi.gov/docs/default-source/default-document-library/pmi-reports/2017-pmi-eleventh-annual-report.pdf?sfvrsn=14.

[63] Bbosa RS, Ehlers VJ (2017). Midwives provision of antimalaria services to pregnant women in Uganda. Midwifery..

[64] Odetola TD, Okanlawon FA (2016). Effects of mHealth nursing intervention on uptake of antenatal care and pregnancy drugs among pregnant women attendees of PHC in Oyo State. J Int Soc Telemed EHealth..

[65] Mbengue MAS, Bei AK, Mboup A, Ahouidi A, Sarr M, Mboup S (2017). Factors influencing the use of malaria prevention strategies by women in Senegal: a cross-sectional study. Malar J..

[66] Nkoka O, Chuang TW, Chen YH (2018). Association between timing and number of antenatal care visits on uptake of intermittent preventive treatment for malaria during pregnancy among Malawian women. Malar J..

[67] Aregbeshola BS, Khan SM (2017). Factors affecting the uptake of malaria prevention strategies among pregnant women in Nigeria: evidence from 2013 Nigeria demographic and health survey. J Public Health..

[68] Jaiteh F, Dierickx S, Gryseels C, O’Neill S, D’Alessandro U, Scott S (2016). ‘Some anti-malarials are too strong for your body, they will harm you.’ Socio-cultural factors influencing pregnant women’s adherence to anti-malarial treatment in rural Gambia. Malar J..

[69] PMI. Burkina Faso: Malaria Operational Plan FY2018. https://www.pmi.gov/docs/default-source/default-document-library/malaria-operational-plans/fy-2018/fy-2018-burkina-faso-malaria-operational-plan.pdf?sfvrsn=5.

[70] PMI. Malawi: Malaria Operational Plan FY2018. https://www.pmi.gov/docs/default-source/default-document-library/malaria-operational-plans/fy-2018/fy-2018-malawi-malaria-operational-plan.pdf?sfvrsn=5.

[71] TIPTOP: advancing prevention of malaria in pregnancy. 2018.

[72] WHO (2007). Malaria in pregnancy. Guidelines for measuring key monitoring and evaluation indicators.

[73] Mutseyekwa F, Mandigo R, Mashizha S, Mukuzunga M, Grand Z, Uzande C (2017). Assessment of facilitators and barriers to achieving the target IPTp Mutasa District, Manicaland Province, Zimbabwe: a formative assessment. Am J Trop Med Hygiene..

[74] Rowe AK, de León GFP, Mihigo J, Santelli ACFS, Miller NP, Van-Dúnem P (2009). Quality of malaria case management at outpatient health facilities in Angola. Malar J..

[75] USAID, PMI. Successes and challenges for malaria in pregnancy programming: a three country analysis. Baltimore: Jhpiego Publ. 2013.

[76] Global call to action to increase national coverage of intermittent preventive treatment of malaria in pregnancy for immediate impact. Roll Back Malaria Partnership, Healthy Newborn Network. 2015.

[77] Rowe AK, de Savigny D, Lanata CF, Victora CG (2005). How can we achieve and maintain high-quality performance of health workers in low-resource settings?. Lancet.

[78] Makene CL, Plotkin M, Currie S, Bishanga D, Ugwi P, Louis H (2014). Improvements in newborn care and newborn resuscitation following a quality improvement program at scale: results from a before and after study in Tanzania. BMC Pregnancy Childbirth..

[79] Manongi RN, Marchant TC, Bygbjerg IbC (2006). Improving motivation among primary health care workers in Tanzania: a health worker perspective. Hum Resour Health.

[80] Johnson P, Fogarty L, Fullerton J, Bluestone J, Drake M (2013). An integrative review and evidence-based conceptual model of the essential components of pre-service education. Hum Resour Health..

[81] Solarin I, Black V (2013). “They Told Me to Come Back”: women’s antenatal care booking experience in inner-city Johannesburg. Maternal Child Health J..

[82] Papageorghiou AT, Ohuma EO, Gravett MG, Hirst J, da Silveira MF, Lambert A (2016). International standards for symphysis-fundal height based on serial measurements from the Fetal Growth Longitudinal Study of the INTERGROWTH-21st Project: prospective cohort study in eight countries. BMJ.

[83] Danhoundo G, Wiktorowicz ME, Yaya S (2017). Governance of malaria prevention: how decision makers’ and pregnant women’s sensemaking contribute to unintended consequences. Health Care Women Int.

[84] Wanzira H, Katamba H, Okullo AE, Rubahika D (2016). The challenge of using intermittent preventive therapy with sulfadoxine/pyrimethamine among pregnant women in Uganda. Malar J..

[85] Ibrahim H, Maya ET, Issah K, Apanga PA, Bachan EG, Noora CL (2017). Factors influencing uptake of intermittent preventive treatment of malaria in pregnancy using sulphadoxine pyrimethamine in Sunyani Municipality, Ghana. Pan Afr Med J..

[86] Ikpeama CA, Ikpeama CJ, Ikpeama OJ, Ogwuegbu JU (2017). Knowledge, attitude and utilization of intermittent preventive treatment for malaria among pregnant women attending antenatal clinic in Usmanu Danfodiyo University Teaching Hospital(UDUTH) Sokoto. Sokoto J Med Lab Sci..

[87] Adeniran A, Goodman OO, Olatona FA, Oluwole EO (2016). Malaria prevention in pregnancy among traditional birth attendants in rural Lagos, Nigeria. J Community Health Primary Care..

[88] WHO (2017). Implementing malaria in pregnancy programs in the context of World Health Organization recommendations on antenatal care for a positive pregnancy experience.

[89] WHO (2018). Malaria surveillance, monitoring & evaluation: a reference manual.

